# C3 Rho-Inhibitor for Targeted Pharmacological Manipulation of Osteoclast-Like Cells

**DOI:** 10.1371/journal.pone.0085695

**Published:** 2013-12-27

**Authors:** Andrea Tautzenberger, Christina Förtsch, Christian Zwerger, Lydia Dmochewitz, Ludwika Kreja, Anita Ignatius, Holger Barth

**Affiliations:** 1 Institute of Orthopaedic Research and Biomechanics, Centre of Musculoskeletal Research, University of Ulm, Ulm, Germany; 2 Institute of Pharmacology and Toxicology, University of Ulm Medical Center, Ulm, Germany; Institute Pasteur, France

## Abstract

The C3 toxins from *Clostridium botulinum* (C3bot) and *Clostridium limosum* (C3lim) as well as C3-derived fusion proteins are selectively taken up into the cytosol of monocytes/macrophages where the C3-catalyzed ADP-ribosylation of Rho results in inhibition of Rho-signalling and characteristic morphological changes. Since the fusion toxin C2IN-C3lim was efficiently taken up into and inhibited proliferation of murine macrophage-like RAW 264.7 cells, its effects on RAW 264.7-derived osteoclasts were investigated. C2IN-C3lim was taken up into differentiated osteoclasts and decreased their resorption activity. In undifferentiated RAW 264.7 cells, C2IN-C3lim-treatment significantly decreased their differentiation into osteoclasts as determined by counting the multi-nucleated, TRAP-positive cells. This inhibitory effect was concentration- and time-dependent and most efficient when C2IN-C3lim was applied in the early stage of osteoclast-formation. A single-dose application of C2IN-C3lim at day 0 and its subsequent removal at day 1 reduced the number of osteoclasts in a comparable manner while C2IN-C3lim-application at later time points did not reduce the number of osteoclasts to a comparable degree. Control experiments with an enzymatically inactive C3 protein revealed that the ADP-ribosylation of Rho was essential for the observed effects. In conclusion, the results indicate that Rho-activity is crucial during the early phase of osteoclast-differentiation. Other bone cell types such as pre-osteoblastic cells were not affected by C2IN-C3lim. Due to their cell-type selective and specific mode of action, C3 proteins and C3-fusions might be valuable tools for targeted pharmacological manipulation of osteoclast formation and activity, which could lead to development of novel therapeutic strategies against osteoclast-associated diseases.

## Introduction

The C3 toxins (~25 kDa) from *Clostridium botulinum* (C3bot1) [1] and *Clostridium limosum* (C3lim) [2] selectively mono-ADP-ribosylate the small guanosine triphosphate (GTP)-binding proteins Rho A, -B, and –C at Asn-41, which inhibits Rho-signalling in mammalian cells [3]. Among a variety of cellular responses, C3-treatment protects cells from apoptosis and inhibits proliferation [3]. Interestingly, C3 toxins are not efficiently taken up into most eukaryotic cell types including epithelial cells and fibroblasts and it was suggested that uptake of C3 toxin into cells might only occur by non-specific pinocytosis when large amounts of C3 are applied for incubation periods longer than 24 h [4]. We discovered recently that monocytes/macrophages are the target cells for the clostridial C3 toxins [5]. These cells internalize comparatively low concentrations of C3 toxins within approx. 3 h, most likely by a specific uptake mechanism including receptor-mediated endocytosis and subsequent translocation from acidified endosomal vesicles into the host cell cytosol [5]. In these cells, the C3-catalyzed Rho-modification leads to re-organization of the actin cytoskeleton and characteristic morphological changes [5]. Enzymatically inactive C3bot1E174Q [6] is internalized into monocytes/macrophages comparable to wild-type C3 proteins [5] and due to lacking adverse effects on cells, it serves as carrier for selective delivery of “foreign” proteins into the cytosol of monocytes/macrophages [7,8]. 

In order to deliver C3 Rho-inhibitor into the cytosol of various cell types, we previously developed the recombinant fusion toxin C2IN-C3lim (~50 kDa), which exploits the binary C2 toxin from *C. botulinum* for its transport into cells [9]. The C2 toxin consists of the actin ADP-ribosylating enzyme component C2I and the separate transport component C2IIa, which delivers C2I into the cytosol of all tested cell types (for review see [10]). The fusion toxin C2IN-C3lim consists of enzymatically active C3lim and the enzymatically inactive N-terminal domain of C2I (C2IN, ~25 kDa) [9]. When applied together with C2IIa, C2IN-C3lim is efficiently delivered into the cytosol of all mammalian cell types tested so far because its C2IN domain interacts with C2IIa and this triggers specific internalization via the C2 toxin uptake pathway [9,11]. However, in the absence of C2IIa, C2IN-C3lim is taken up into monocytes/macrophages but not into other cell types [5]. Like the clostridial C3 toxins, C2IN-C3lim is selectively taken up into macrophage-like cells by the C3-specific uptake mechanism via acidified endosomal vesicles [5,9]. Regarding its Rho-selective ADP-ribosyltransferase activity and the cellular effects, C2IN-C3lim behaves like C3lim [9]. 

Since C3 toxins are the only known Rho-inhibitors and selectively target cells from the monocyte/macrophage-line, C3 toxins and C3-derived fusion toxins such as C2IN-C3lim are ideal tools for investigation and targeted pharmacological manipulation of Rho-dependent signal transduction in cells which are related to this cell line such as osteoclasts. *In vivo*, monocytes/macrophages and osteoclasts are derived from pluripotent hematopoietic stem cells [12] and *in vitro*, osteoclasts differentiate from macrophage-like RAW 264.7 cells. Osteoclasts form a tight sealing zone on mineralized surfaces which is essential for resorption of matrix, e.g. during re-organization of bone tissue [13]. It was reported earlier that the C3-catalyzed ADP-ribosylation of Rho in osteoclasts resulted in disruption of their sealing zone and their resorption activity [14], indicating that Rho plays a crucial role for osteoclast activity. Here, we demonstrate that C2IN-C3lim is efficiently taken up into the cytosol of RAW 264.7 cells and derived osteoclasts. Treatment with C2IN-C3lim inhibited the resorption activity of already differentiated osteoclasts but also the formation of osteoclasts from RAW 264.7 cells due to the C3-catalyzed ADP-ribosylation of Rho. In contrast, C2IN-C3lim had no effects on other cultured bone cell types such as murine pre-osteoblastic MC3T3 cells, confirming its monocyte/macrophage-selective mode of action.

## Results

### Selective and specific uptake of C2IN-C3lim into the cytosol of RAW 264.7 cells

**Figure 1:**
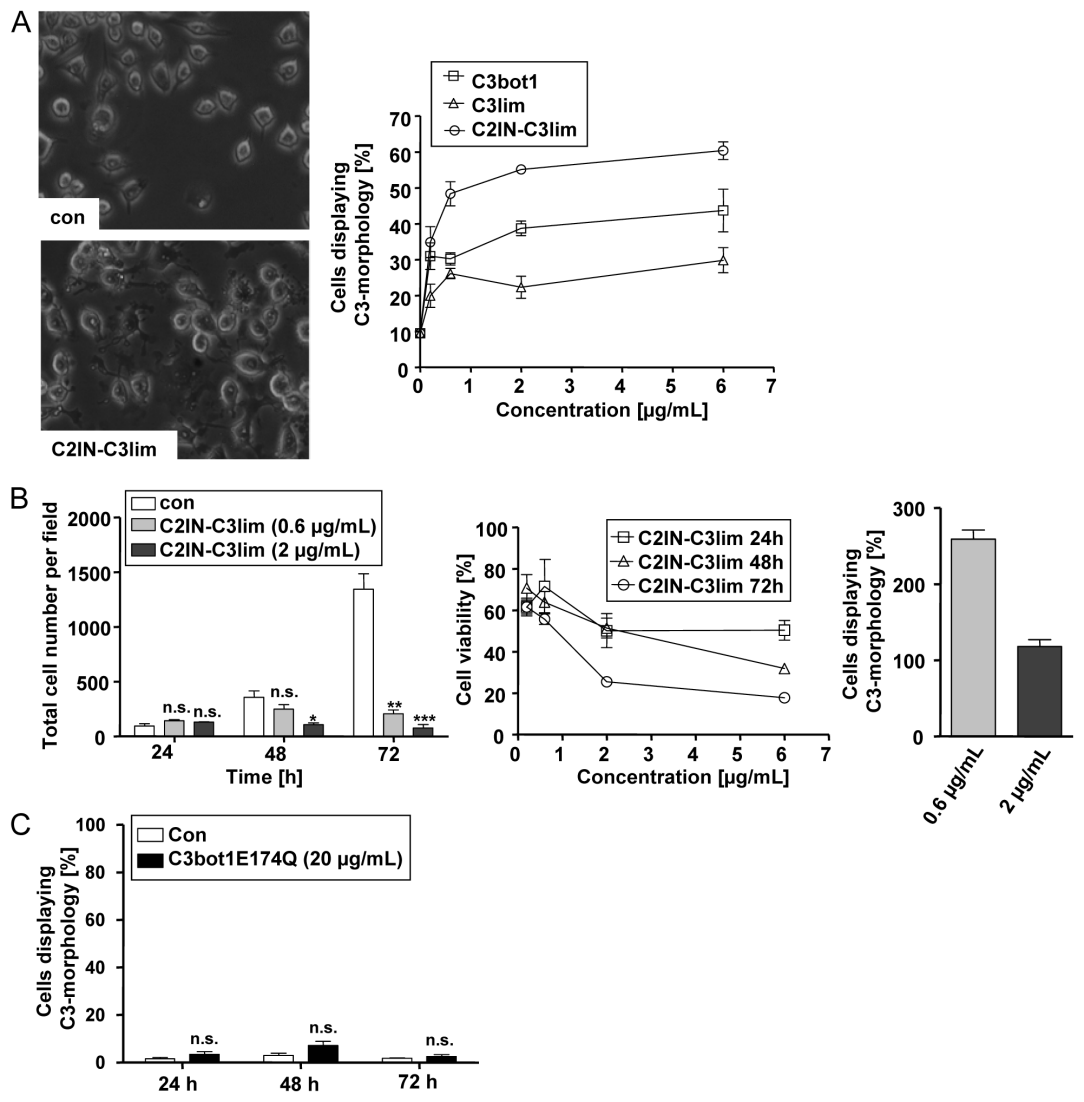
Effect of C3bot1, C3lim and C2IN-C3lim on RAW 264.7 cells. *A*. Effect of C3bot1, C3lim and C2IN-C3lim on the morphology of RAW 264.7 cells grown in 12 well plates. Each of the proteins were added in final concentrations of 0.2 µg/mL (= 4 nM C2IN-C3lim, 8 nM C3bot1 or C3lim), 0.6 µg/mL (= 12 nM C2IN-C3lim, 24 nM C3bot1 or C3lim), 2 µg/mL (= 40 nM C2IN-C3lim, 80 nM C3bot1 or C3lim), and 6 µg/mL (= 120 nM C2IN-C3lim, 240 nM C3bot1 or C3lim) into the medium and cells were incubated in the presence of the proteins and pictures were taken from the cells after 24 h. For control, cells were left untreated. The morphology of control cells and C2IN-C3lim-treated cells is shown in the left panel. The percentages of cells displaying the typical “C3-morphology” were calculated from six individual pictures (right panel). The values were given as mean ± S.D. (n = 6). *B*. Effect of C2IN-C3lim on the proliferation of RAW 264.7 cells. Cells were incubated in 96 well plates with 0.6 and 2 µg/mL C2IN-C3lim or left untreated for control. After 24, 48 and 72 h pictures from the cells were taken and the total number of cells determined from 3 different pictures (left panel). The values were given as mean ± S.D. (n = 3). Significance was determined by student’s t-test for cells treated with the respective C3 protein against untreated (*** = p<0.001; ** = p<0.01; * = p<0. 1; n.s. = not significant). Alternatively, RAW 264.7 cells grown in 96 well plates were incubated with 0.6 and 2 µg/mL C2IN-C3lim or left untreated for control. After 24, 48 and 72 h the amount of viable cells was determined by MTT test (mid panel). The values are given in percent from control cells as mean ± S.D. (n = 3). Right panel: The values for 72 h C2IN-C3lim are given in percent from the values after 24 h C2IN-C3lim-treatment with the respective concentration of C2IN-C3lim. *C*. Effect of C3bot1E174Q on the morphology of RAW 264.7 cells. RAW 264.7 cells grown in 96 well plates were incubated for up to 72 h with 20 µg/mL (= 800 nM) C3bot1E174Q or left untreated for control. Pictures from the cells were taken and the percentages of cells displaying “C3-morphology” calculated from three individual pictures. The values were given as mean ± S.D. (n = 3). Significance was determined by student’s t-test for cells treated with C3bot1E174Q against untreated (n.s. = not significant).

**Figure 2:**
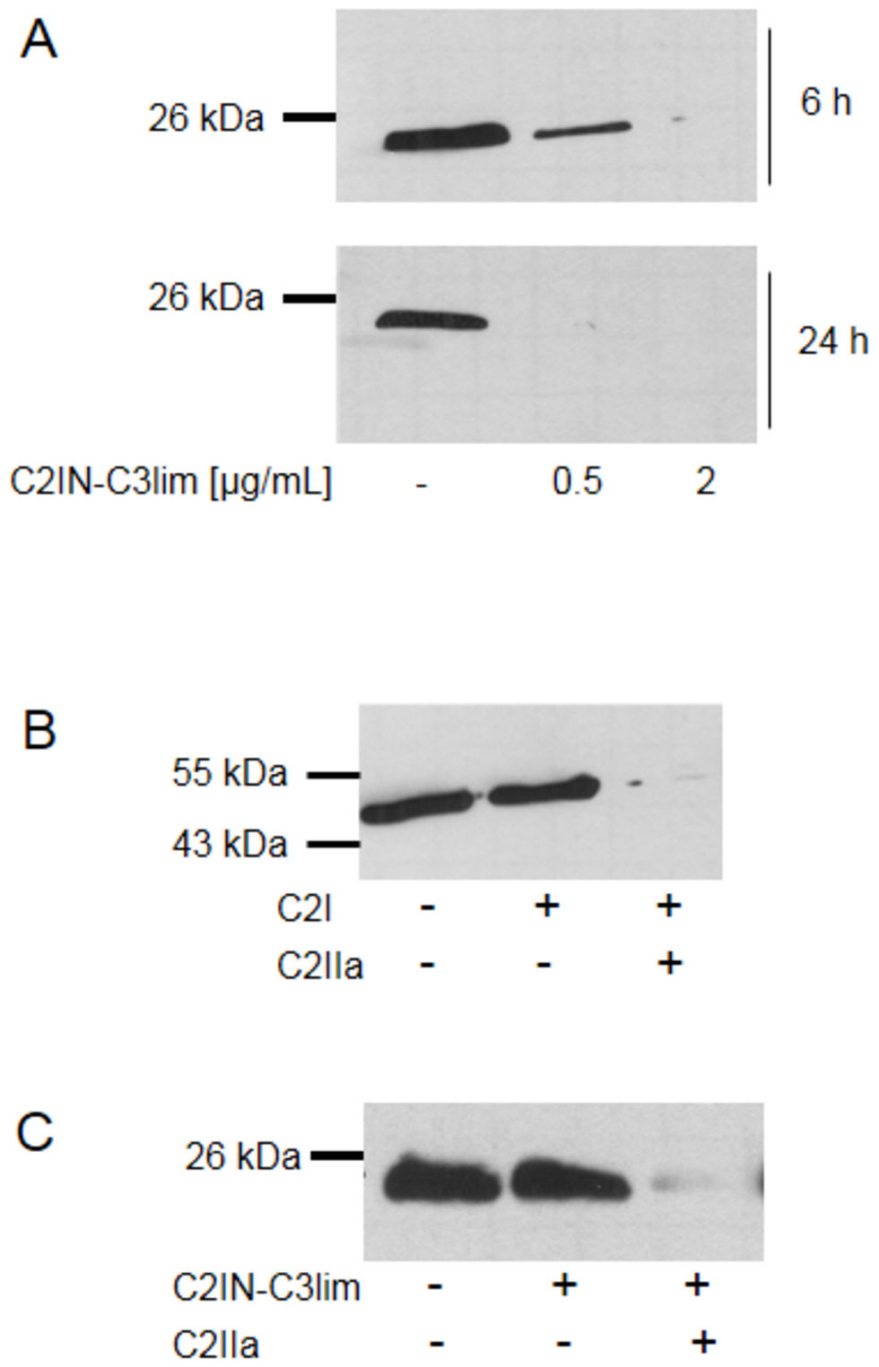
Specific and selective uptake of C2IN-C3lim into macrophage-like RAW 264.7 cells. *A*. ADP-ribosylation status of Rho in RAW 264.7 cells treated with C2IN-C3lim. Cells were incubated with 0.5 or 2 µg/mL of C2IN-C3lim or left untreated for control. The cells were lysed after 6 and 24 h and equal amounts of lysate proteins incubated with fresh C3bot1 and biotin-labelled NAD^+^. The biotinylated, i.e. ADP-ribosylated Rho is shown. Equal amounts of loaded protein were confirmed by Ponceau S staining of the blotted proteins (not shown). *B*. C2I alone is not taken up into RAW 264.7 cells. Cells were incubated with C2I (2 µg/mL) alone or with C2I (0.4 µg/mL) + C2IIa (0.8 µg/mL) or left untreated for control. After 6 h cells were lysed and equal amounts of lysate proteins incubated with fresh C2I and biotin-labelled NAD^+^. The biotinylated, i.e. ADP-ribosylated actin is shown. Equal amounts of loaded protein were confirmed by Ponceau S staining of the blotted proteins (not shown). *C*. C2IN-C3lim is not taken up into pre-osteoblastic MC3T3 cells under comparable conditions. Cells were incubated with C2IN-C3lim (5 µg/mL) or with C2IN-C3lim (1 µg/mL) + C2IIa (2 µg/mL) or left untreated for control. After 6 h the cells were lysed and the ADP-ribosylation status of Rho determined as described in A. The biotinylated, i.e. ADP-ribosylated Rho is shown. Equal amounts of loaded protein were confirmed by Ponceau S staining of the blotted proteins (not shown).

When the effect of increasing concentrations of C3bot1, C3lim and C2IN-C3lim on the morphology of RAW 264.7 was compared after a 24 h treatment, C2IN-C3lim was most efficient (Figure 1A). Treatment of RAW 264.7 cells with C2IN-C3lim resulted in a decreased number of cells after 2 and 3 days of incubation (Figure 1B, left panel). When the viable cells were determined by MTT assay, there was a decreased amount after C2IN-C3lim-treatment in comparison to untreated cells (Figure 1B, mid panel). However, the amount of viable cells was not decreased in C2IN-C3lim-treated cells from 24 to 72 h (Figure 1B, right panel), suggesting that C2IN-C3lim inhibited proliferation but did not induce death of RAW 264.7 cells. Treatment of cells with 20 µg/mL (= 800 nM) of the enzymatic inactive C3botE174Q had no effect on the morphology (Figure 1C) and viability (not shown) of RAW 264.7 cells, even not after 3 days of incubation, implicating that C3-catalyzed ADP-ribosylation of Rho was essential for the observed effects of C3bot1, C3lim and C2IN-C3lim on RAW 264.7 cells. Therefore, the uptake of C2IN-C3lim into the cytosol of RAW 264.7 cells was analyzed by investigating the ADP-ribosylation status of Rho from these cells. Cells were incubated with 0.5 µg/mL (= 10 nM) and 2 µg/mL (= 40 nM) of C2IN-C3lim in the medium or left untreated for control. After 6 and 24 h the cells were lysed and the ADP-ribosylation status of Rho was determined from their lysates by sequential ADP-ribosylation. As shown in Figure 2A, untreated control cells show strongly biotin-labelled Rho while lysates from the C2IN-C3lim-treated cells show much weaker or no signals. In these cells, the major portion of Rho was already ADP-ribosylated during incubation of the living cells with C2IN-C3lim and this already modified Rho did not serve as substrate for the subsequent *in vitro* ADP-ribosylation. Taken together, the results indicate that comparatively low amounts of C2IN-C3lim ADP-ribosylated Rho in RAW 264.7 cells implying the efficient uptake of C2IN-C3lim into their cytosol within 6 h. 

Importantly, the uptake of C2IN-C3lim into the cytosol was specifically mediated by its C3 moiety since C2I was not taken up into RAW 264.7 cells as confirmed by sequential ADP-ribosylation of actin from lysates of these cells (Figure 2B). In contrast to C2IN-C3lim, C2I was only taken up into the cytosol of RAW 264.7 cells when applied in combination with the separate transport component C2IIa but not alone. 

Prompted by these results, the uptake of C2IN-C3lim into other bone cell types such as murine pre-osteoblastic MC3T3 cells was tested by the same approach. In contrast to RAW 264.7, these cells did not internalize C2IN-C3lim into their cytosol as confirmed by sequential ADP-ribosylation of Rho from the lysates of these cells (Figure 2C). However, when applied together with C2IIa, C2IN-C3lim was taken up into MC3T3 cells, indicating that its C3 portion ADP-ribosylated Rho when C2IN-C3lim is delivered by an alternative mechanism into the cytosol (Figure 2C). In conclusion, C2IN-C3lim is efficiently and selectively internalized into and inhibits proliferation of cells of the osteoclastic RAW 264.7. Therefore C2IN-C3lim can be used to investigate effects of C3-catalyzed Rho-inhibition on activity and differentiation of osteoclasts derived form RAW 264.7 cells.

### C2IN-C3lim inhibits osteoclast-formation from RAW 264.7 cells in a time- and concentration-dependent manner due to the ADP-ribosylation of Rho

**Figure 3:**
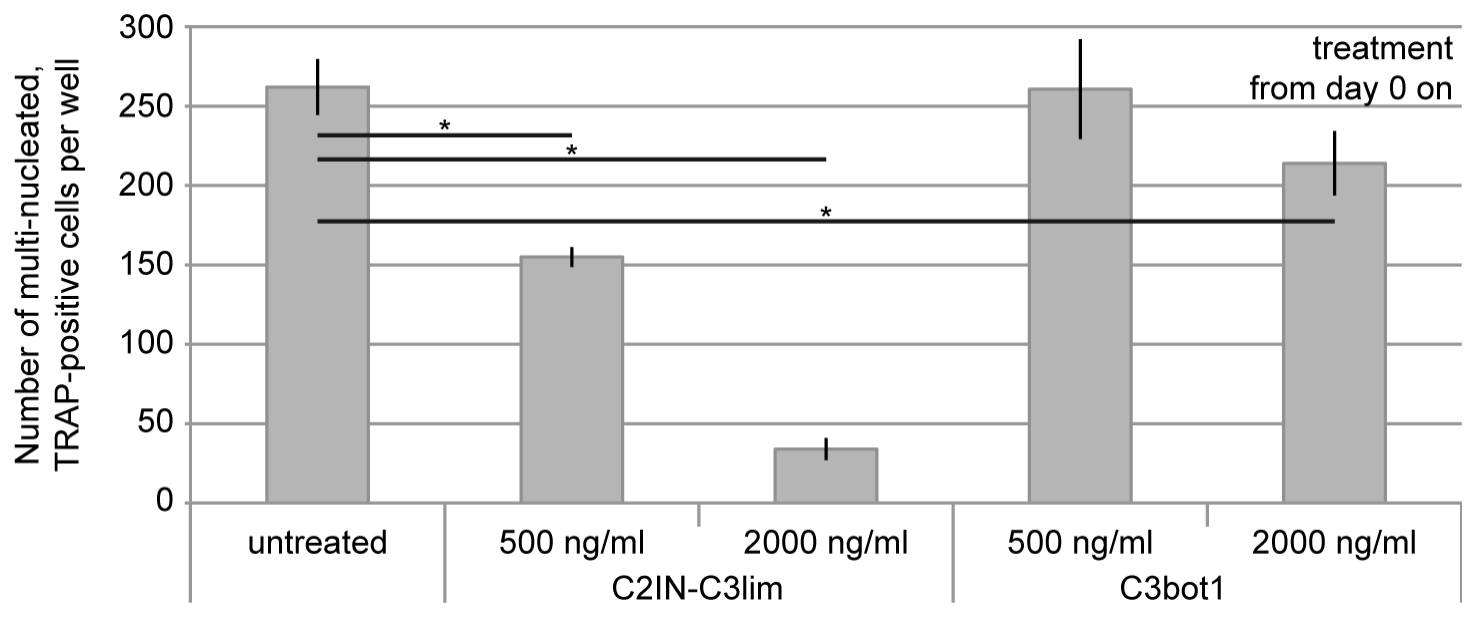
Inhibition of osteoclast-formation by C3-treatment. C3bot1 or C2IN-C3lim (0.5 and 2µg/mL) were added from day 0 on to RAW264.7 cells. For control, cells were left untreated. RANKL was added and the number of multi-nucleated and TRAP-positive osteoclasts per well (96 well plate) was counted at day 5.

**Figure 4:**
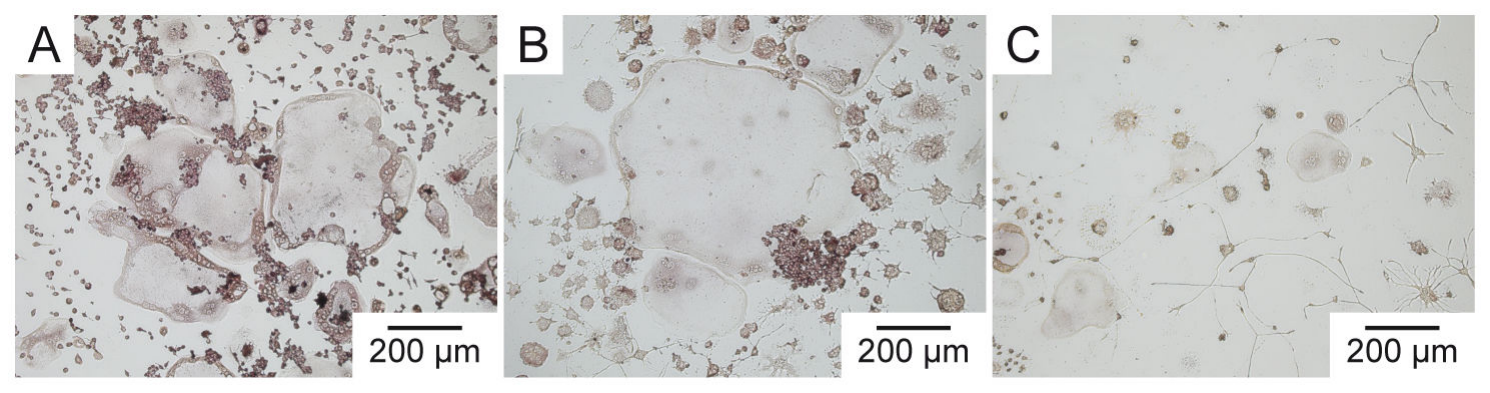
Effect of C2IN-C3lim on osteoclast-formation. C2IN-C3lim (0.5 and 2µg/mL) was added to RAW264.7 cells from day 0 on and cells were treated with RANKL. For control, cells were treated with RANKL in the absence of C2IN-C3lim. At day 5, osteoclasts were stained for tartrate-resistant acid phosphatase. Osteoclasts formed in the absence (A) and presence of increasing concentration of C2IN-C3lim (B: 0.5 µg/mL; C: 2 µg/mL) are shown.

The RANKL (receptor activator of nuclear factor‑κB ligand)-induced formation of osteoclasts from RAW 264.7 cells was investigated in the presence and absence of the Rho-ADP-ribosylating C3 toxin. To this end, RAW 264.7 cells were incubated for 5 days with 0.5 µg/mL and 2 µg/mL of C3bot1 or C2IN-C3lim in the medium and osteoclast-formation was determined by counting the multi-nucleated and TRAP-positive cells after this period. As shown in Figure 3, C3-treatment from day 0 on resulted in a concentration-dependent decrease of osteoclast-formation and the inhibitory effect was stronger for C2IN-C3lim. Therefore, C2IN-C3lim was used for further experiments. Figure 4A shows the morphology of RAW 264.7 cells cultured in the absence of C2IN-C3lim. Here, numerous multi-nucleated, positively stained osteoclasts were formed during the differentiation of RAW 264.7 cells. In contrast, treatment with C2IN-C3lim reduced the number of multi-nucleated, TRAP-positive osteoclasts in a concentration-dependent manner (Figure 4B and C) and the C3-treated cells formed pronounced cellular protrusions. 

**Figure 5:**
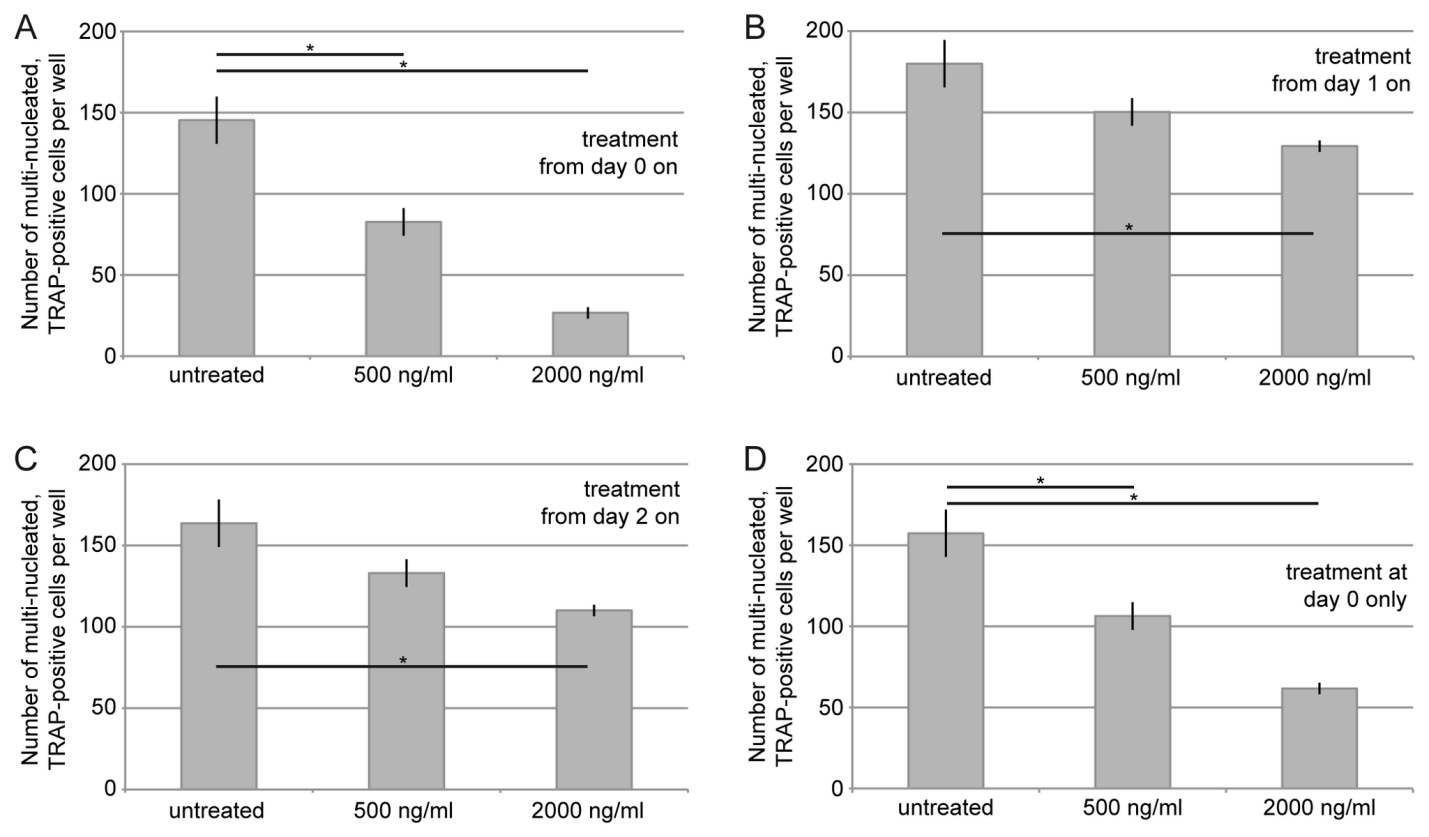
Application of C2IN-C3lim in the early stage of differentiation inhibits osteoclast-formation. C2IN-C3lim (0.5 and 2µg/mL) was added from day 0 on (A), from day 1 on (B), from day 2 on (C) or at day 0 only with subsequent medium change on day 1 (D). The number of multi-nucleated (at least three nuclei) and TRAP-positive osteoclasts per well (96 well plate) were determined at day 5.

To investigate the inhibitory effect of C3-treatment on osteoclast-formation in more detail, it was tested whether the time point of C3-application was crucial to inhibit osteoclast-formation. To this end C2IN-C3lim was added to RANKL-treated RAW 264.7 cells either from day 0 on (Figure 5A), or from day 1 on (Figure 5B), or from day 2 on (Figure 5C). For control, cells were incubated with RANKL but without C2IN-C3lim. At day 5 the number of multi-nucleated, TRAP-positive cells was determined. The strongest inhibitory effect on osteoclast-formation was observed when C2IN-C3lim had been added from day 0 on, i.e. in the early stage of osteoclast-differentiation (Figure 5A). Application of C2IN-C3lim from later points in time on (Figure 5B and C) did not reduce the number of multi-nucleated, TRAP-positive cells to a comparable degree. Furthermore, a single-pulse incubation with C2IN-C3lim only at day 0 and subsequent removal of C2IN-C3lim from the cultures by medium change at day 1 (Figure 5D) led to a comparable inhibitory effect as a continuous C2IN-C3lim-incubation from day 0 on (Figure 5A). These results confirm that the early stage of differentiation is crucial for unimpeded osteoclast-formation and imply that the C3-catalyzed inhibition of Rho-signalling only at day 0 is sufficient to inhibit osteoclast-formation. 

### C3-treatment reduces the resorption activity of differentiated osteoclasts

**Figure 6:**
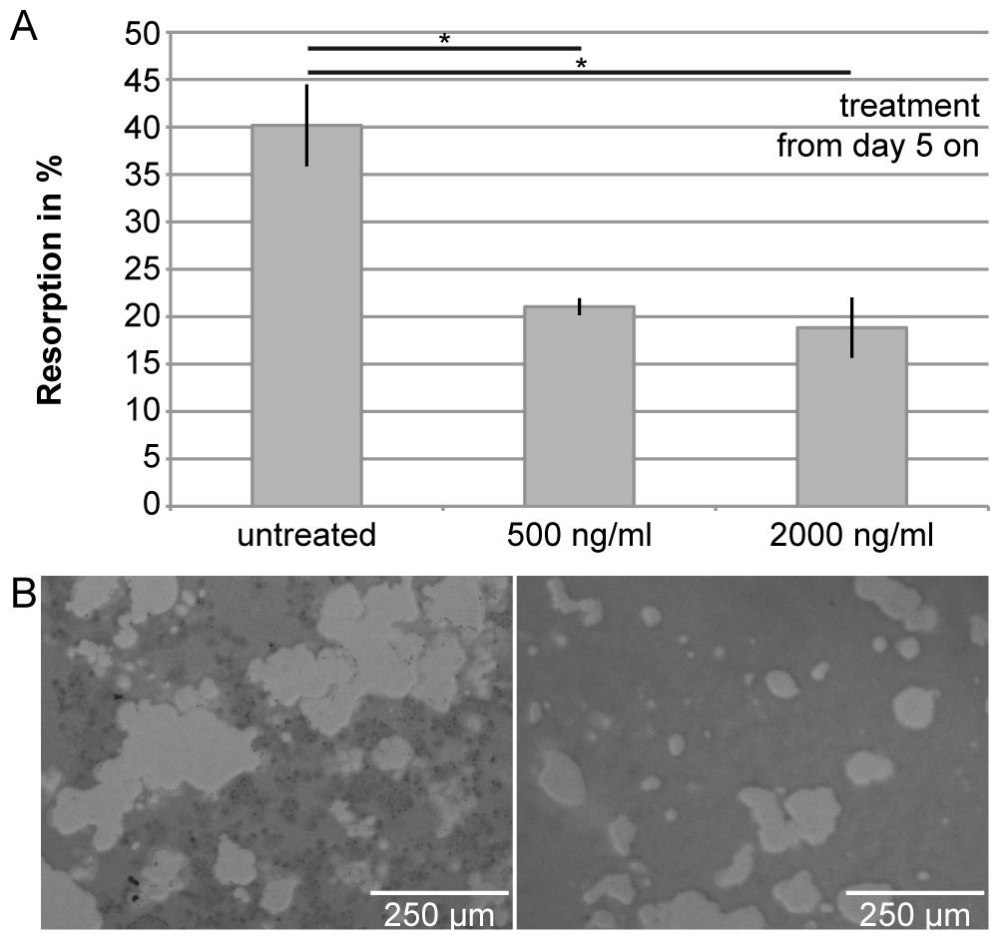
Treatment with C2IN-C3lim decreases the resorption of synthetic calcium phosphate surfaces by already differentiated osteoclasts. C2IN-C3lim (0.5 and 2µg/mL) was added to osteoclasts growing on synthetic calcium phosphate surfaces from day 5 on, i.e. after undisturbed osteoclast-differentiation from RAW264.7 cells. For control, no C2IN-C3lim was added. At day 13, resorption activity of osteoclasts was determined by quantification of the resorbed area per well (A). Qualitative images of resoption areas on sythetic calcium phosphate surfaces are shown below (B, left: control; right: C2IN-C3lim (2µg/mL)).

The effect of C3-treatment on fully differentiated osteoclasts regarding their resorption activity was investigated. RAW 264.7-derived osteoclasts were grown on synthetic calcium phosphate surfaces and treated with C2IN-C3lim from day 5 on, i.e. after the formation of multi-nucleated osteoclasts, to clearly distinguish between toxin effects on osteoclast-formation and activity. Quantification of the resorbed areas after 13 days of culture revealed a significantly reduced resorption by osteoclasts after C3-treatment (Figure 6). Again, treatment of the cells with enzymatically inactive C3bot1E174Q had no effect on the resorption by osteoclasts (data not shown), indicating that the C3-catalyzed Rho-modification in the cytosol of the osteoclasts was crucial for this effect.

### Internalization of C2IN-C3lim into differentiating osteoclasts induces characteristic morphological changes

**Figure 7:**
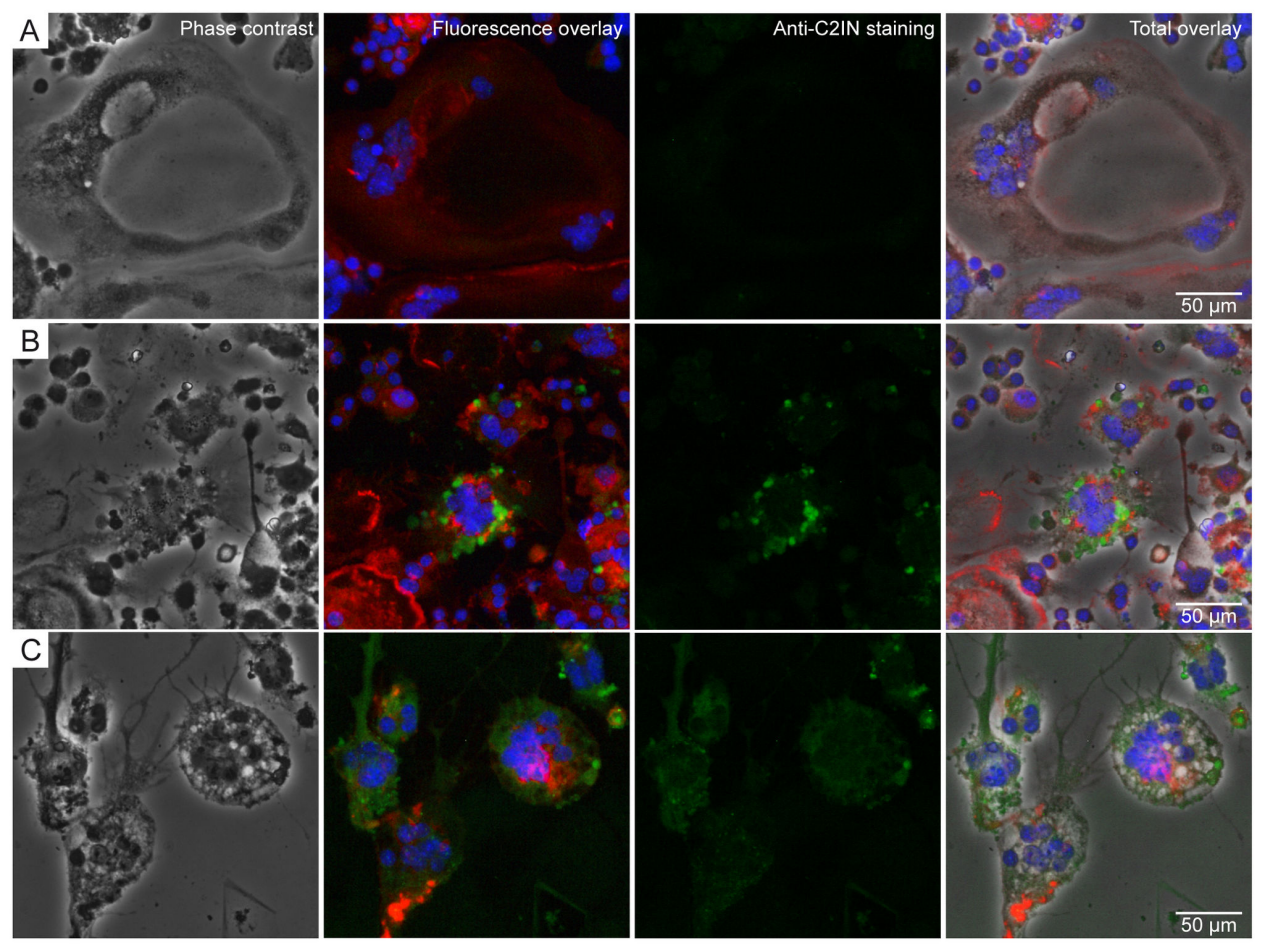
Uptake of C2IN-C3lim into differentiating osteoclasts and morhophological changes caused by C2IN-C3lim. RAW264.7 cells which were grown in the presence of RANKL to induce differentiation to osteoclasts. Cells were left untreated for control (A) or treated with C2IN-C3lim (2 µg/mL) at day 0 and 2 (B and C). At day 5, osteoclasts were stained for actin (red), nuclei (blue) and C3 (green) and cells analyzed by phase contrast microscopy (left row) and fluorescence microscopy.

Finally, the uptake of C2IN-C3lim into differentiating osteoclasts and the morphological changes induced by C3-treatment were analyzed by immunofluorescence microscopy. RAW 264.7 cells cultured in the absence of C2IN-C3lim with RANKL formed cells with multiple nuclei (Figure 7A, blue staining). Moreover, their actin cytoskeleton was organised in a seemingly ring-like structure as mainly anticipated for potentially active osteoclasts (Figure 7A, red staining). Cells cultured in the presence of C2IN-C3lim showed a characteristic change of their morphology (Figure 7B and C) including shrinking of the cell bodies and formation of multiple thin protrusions accompanied by re-organization of the actin cytoskeleton, which is characteristic for C3-treated cells. The typical morphological changes indicate that enzymatically active C3 was present in the cytosol of these cells. 

The detection of cell-associated C2IN-C3lim with specific C3-antiserum confirmed the C3-uptake into differentiating osteoclasts (Figure 7B and C, green staining), whereas no C3-specific staining was detected in untreated control cells (Figure 7A). The images of C2IN-C3lim-treated osteoclasts (Figure 7C) show the distribution of internalized C2IN-C3lim in the cells. The punctual green staining (Figure 7B) likely indicated distinct localisation of C2IN-C3lim in endosomal vesicles while the more diffuse distribution of the green staining (Figure 7C) might represent C2IN-C3lim which had already been released from the endosomal vesicles into the cytosol. The distribution of the green staining over the whole cell bodies including the protrusions suggested an extensive uptake of C2IN-C3lim (Figure 7C). However although C2IN-C3lim alone was taken up into differentiating osteoclasts in a sufficient amount to induce cellular effects, its uptake into the cytosol of osteoclasts was enhanced when the separate transport component C2IIa was added (data not shown). 

## Discussion

Prompted by our earlier findings that clostridial C3bot1 and C3lim toxins are selectively taken up by cells of the monocyte/macrophage line [5], we have performed a series of experiments to investigate the effects of C3-treatment on osteoclasts which were generated by RANKL-induced differentiation of murine osteoclastic RAW 264.7 cells. Like the clostridial C3 toxins, the recombinant C2IN-C3lim fusion toxin [9] was selectively internalized into undifferentiated RAW 264.7 cells and already differentiated osteoclasts by the C3-specific uptake mechanism. Interestingly, C2IN-C3lim exhibited a stronger effect than C3lim alone or C3bot on undifferentiated RAW 264.7 cells. Although the reason for this unexpected effect is not known, one could speculate that the C2IN portion enhances the uptake of the C3 ADP-ribosyltransferase into the cytosol of the macrophages. In particular, C2IN could enhance the transport of internalized C2IN-C3lim protein across endosomal membranes from the endosomal lumen into the cytosol [5] since C2IN mediates this translocation step of the C2I ADP-ribosyltransferase through C2IIa-pores across endosomal membranes [9]. Moreover, C2IN could serve as a scaffold protein which may facilitate refolding of C3lim in the cytosol if an unfolding of C3lim is required for membrane translocation, which is not clear so far. Therefore, C2IN-C3lim was used to investigate the effects mediated by C3-catalyzed Rho-inhibition in differentiating osteoclasts and in already differentiated osteoclasts. 

By using this fusion toxin, we confirmed earlier results by another group that C3-catalyzed Rho inhibition in already differentiated osteoclasts decreases the resorption activity of these cells [14]. It was reported by various groups that Rho activity regulates the formation of the actin ring in osteoclasts which is a prerequisite for bone resorption by these cells [14–16]. Moreover, we discovered that application of C2IN-C3lim to RAW 264.7 cells inhibited their RANKL-induced differentiation into osteoclasts in a time- and concentration-dependent manner which might be a consequence of the inhibited proliferation of C2IN-C3lim-treated RAW 264.7 cells. A weaker inhibitory effect on osteoclast-differentiation was observed when C3bot1 was used instead of C2IN-C3lim while enzymatically inactive C3bot1E174Q [6] had no effect on the morphology of RAW 264.7 cells. Moreover, these results confirmed that C2IN-C3lim is an attractive tool to investigate the specific C3-mediated effects in such cells. The concentration- and time-dependent inhibition of osteoclast-formation by C2IN-C3lim with the strongest effect after a single-dose of C2IN-C3lim at day 0 or C2IN-C3lim-treatment from day 0 on implies an essential role of Rho in the early phase of osteoclast-differentiation. Although the results imply that a time-dependent Rho-inhibition seems to be crucial for osteoclast-formation, the reason for this effect is not known so far. Interestingly, in contrast to RhoU, the expression of RhoA, -B and –C, which are the selective targets of C3 proteins is not upregulated during RANKL-induced osteoclastogenesis [17]. However, it is not clear whether the merely constant expression of RhoA, -B, and -C over time is related to the strong effect that is exerted by C3 in early osteoclast differentiation. 

Besides its role as a specific inhibitor to investigate the role of Rho-signalling in osteoclastogenesis and osteoclast functions, the finding that C2IN-C3lim is taken up into the cytosol of osteoclasts but not of other bone cell types such as pre-osteoblastic cells might have a pharmacological impact. The observation that C3-derived recombinant fusion toxins such as C2IN-C3lim are taken up into osteoclasts is an essential prerequisite for exploiting enzymatically inactive C3 protein such as C3bot1E174Q as transport systems for targeted delivery of pharmacologically active molecules including siRNA into osteoclasts for targeted manipulation of osteoclast functions [18,19] *in vitro* and *in vivo*. We have recently demonstrated that C3bot1E174Q selectively delivers proteins and enzymes into cultured macrophages including primary human macrophages derived from monocytes from blood donors [7,8,20]. Because C3-based transporters target monocytes/macrophages in general, they would not serve for a selective drug delivery into osteoclasts after a systemic application. However, a targeted local application of either wild-type C3 for Rho-inhibition in osteoclasts or C3-derived transport systems for targeted drug delivery into osteoclasts might be an appropriate approach to manipulate osteoclastogenesis and/or osteoclast functions, e.g. to improve the osseous integration of orthopaedic implants by suppressing osteoclast activity at the implant surface [21]. Local application in bone and controlled release of C3 proteins or C3-transporters from orthopaedic implant surfaces could be achieved by the use of biocompatible carriers such as resorbable polymers or hydrogels.

## Materials and Methods

### Materials and reagents

Cell culture materials were from TPP (Trasadingen, Switzerland). Dulbecco’s Modified Eagle’s Medium (DMEM) was from LGC Standards GmbH (Wesel, Germany) and alpha‑minimal essential medium (alpha-MEM) from Biochrom (Berlin, Germany). Foetal calf serum (FCS) and L‑glutamine were from PAA Laboratories GmbH (Cölbe, Germany). Hoechst 33342, penicillin‑streptomycin, Alexa 488-coupled goat anti-rabbit antibody and Alexa 594-coupled phalloidin were from Invitrogen (Karlsruhe, Germany). Murine recombinant receptor activator of nuclear factor‑κB ligand (RANKL) and biotinylated NAD^+^ was from R&D Systems GmbH (Wiesbaden-Nordenstadt, Germany). Acid Phosphatase, Leukocyte (TRAP) Kit for osteoclast staining and Triton X-100 were from Sigma-Aldrich (Munich, Germany). Complete® protease inhibitor and streptavidin-peroxidase were from Roche (Mannheim, Germany). Page Ruler pre-stained Protein ladder^®^ was from Fermentas (St. Leon Rot, Germany). Anti-C2IN and anti-C3bot1E174Q antisera were raised in rabbits from Pineda (Berlin, Germany). Enhanced chemiluminescence (ECL) system was obtained from Millipore (Schwalbach, Germany). Nitrocellulose blotting membrane was from Whatman^®^ (Dassel, Germany). Thrombin was from Amersham Biosciences Europe GmbH (Freiburg, Germany). Alexa-488 coupled antibody and phalloidin-Alexa 594 coupled conjugate were purchased from Invitrogen (Karlsruhe, Germany). The recombinant proteins C3lim, C3bot1, C3bot1E174Q, C2IN-C3lim, C2I and C2IIa were expressed as GST-tagged proteins in *E.coli* BL21 and purified by affinity chromatography as described previously [5,8,9]. 

### Cell culture

Murine macrophage-like RAW 264.7 cells (from LGC Standards GmbH/American Type Culture Collection, Wesel, Germany) were grown at 1,500 cells/cm^2^ in DMEM containing 10 % FCS, 1 % L‑glutamine and 1 % penicillin‑streptomycin at 37 °C, 5 % CO_2_ and saturated humidity for up to 13 days depending on the experimental setting. To support the differentiation of RAW 264.7 cells into osteoclasts *in vitro* [22], the medium was supplemented with 40 ng/mL of RANKL. Cells of the murine pre-osteoblastic MC3T3-E1 cell line (German Collection of Microorganisms and Cell Cultures, Braunschweig, Germany) were seeded at 10,000 cells/cm^2^ in alpha‑minimal essential medium (Biochrom AG, Germany) with 10 % FCS, 1 % L‑glutamine and 1 % penicillin‑streptomycin and were cultured at 37 °C, 5 % CO_2_ and saturated humidity for up to 2 days. C3 toxin was added for 6 h and cells cultured in the absence of toxin were used as control. To investigate the effects of C3bot1, C3lim or C2IN-C3lim on undifferentiated RAW 264.7 cells, cells were treated for the indicated time periods with the indicated concentrations of the individual C3 proteins in the medium. To analyze the C3-induced changes in cell morphology, pictures from the cells were taken by using an Olympus IC70 24 light microscope with a C-5060 camera (Olympus, Hamburg, Germany).

### MTT assay

The MTT-assay was performed essentially as described by Alley et al. [23]. Briefly, 7,000 cells/well were incubated for 4 h in 96-well plates at 37 °C in 5% CO_2_. 100 µL of medium (control) or medium containing various concentrations of toxin were added to each well with 3-well replicates per concentration. The cells were incubated for 24, 48 and 72 h. At the end of the incubation period, 50 µL/well of MTT (3-[4,5-dimethylthiazol-2-yl]-2,5-diphenyltetrazolium bromide; Sigma-Aldrich, Munich, Germany) at 1 mg/mL in PBS was added. After 4h, medium was aspirated and formazan crystals were dissolved in dimethyl sulfoxide (DMSO; Merck, Darmstadt, Germany). The light absorbance was measured using a spectrophotometer (Tecan, Crailsheim, Germany) at a wavelength of 570 nm (test) and 650 nm (reference), respectively. 

### Effect of C3-treatment on osteoclast-formation

To assess the influence of C3-treatment on osteoclast-formation, C3 was administered to RAW 264.7 cells during their differentiation to osteoclasts from day 0 on (i.e. at day 0, 1 and 2 of culture), from day 1 on (i.e. at day 1 and 2 of culture), from day 2 on (i.e. at day 2 of culture), or at day 0 only (with subsequent medium change at day 1) to analyze time-dependent effects more closely. After 5 days of culture in 96 well plates, differentiated osteoclasts were stained with the Acid Phosphatase, Leukocyte (TRAP) Kit according to the manufacturer’s instructions to verify the presence of tartrate-resistant acid phosphatase (TRAP), an enzyme specific for the monocyte/macrophage lineage. Briefly, incubation with a TRAP substrate resulted in a red staining. TRAP-positive cells with at least three nuclei were counted as osteoclasts at 400‑fold magnification in order to quantitatively determine the formation of multi‑nucleated cells. Images of the stained cells were taken with a Leica DMI6000 B microscope and a DFC420 C camera (Leica Microsystems, Mannheim, Germany).

### Effect of C3-treatment on osteoclast-activity

The resorption activity of osteoclasts was assessed in cell culture plates with a synthetic calcium phosphate coating (Osteo Assay Surface; Corning Life Sciences, Amsterdam, The Netherlands). C3 was added after osteoclast-formation from day 5 on, i.e. at day 5 and 9 of the experiment. At day 13, the culture plates were filled with 6 % bleach for 5 min to detach the cells and washed twice with pure water. Non-resorbed surface appeared light brown, resorbed regions appeared transparent. The resorption area was quantified using the image processing software MetaMorph AF, version 1.4.0 (Leica Microsystems, Mannheim, Germany).

### Analysis of C3 uptake into cells by immunofluorescence

C3 was added at day 0 and 2 to RAW 264.7 cells which were differentiating into osteoclasts and intracellular C3 was stained at day 5. Cells were fixed with 2 % paraformaldehyde (PFA) containing Hoechst 33342 (1:5,000) for 25 min and incubated with 100 mM glycine for 5 min to inactivate PFA. Cells were permeabilized with 0.2 % Triton X-100 in phosphate buffered saline (PBS) for 5 min and blocked with 4 % bovine serum albumin in PBS with 0.1 % Tween 20 for 45 min at 37 °C. Samples were incubated with anti-C2IN antiserum for 45 min at 37 °C. As a secondary antibody, an Alexa 488-coupled goat anti-rabbit antibody (1:2,000) was used and the actin cytoskeleton was visualised via subsequent incubation with Alexa 594-coupled phalloidin (1:200) for 30 min at 37 °C. Fluorescence microscopy was performed with the DMI6000 B microscope and a DFC360 FX camera (Leica Microsystems, Mannheim, Germany).

### Sequential ADP-ribosylation of Rho in lysates from RAW 264.7 cells

To analyze the ADP-ribosylation status of Rho from RAW 264.7 cells, cells were lysed and 10 µg of whole lysate protein incubated for 30 min at 37 °C with biotin-labelled NAD^+^ (10 µM) and 300 ng of C3bot1 in a buffer containing 20 mM Tris-HCl (pH 7.5), 1 mM EDTA, 1 mM DTT, 5 mM MgCl_2_, complete^®^ protease inhibitor. The reaction was stopped by adding 5 x SDS-sample buffer (625 mM Tris/HCl pH 6.8, 20 % SDS, 8.5 % glycerol, 0.2 % bromophenol blue, 100 mM DTT) and heating for 10 min at 95 °C. Subsequently, the ADP-ribosylated proteins were analyzed by Western blotting. 

### SDS-PAGE and Western blotting

Equal amounts of RAW 264.7 lysate protein were separated by SDS-PAGE [24] and transferred to a nitrocellulose membrane. After blocking for 30 min with 5 % dry milk in PBS containing 0.1 % Tween-20 (PBS-T), the biotin-labelled Rho or actin was detected with streptavidin-peroxidase by using a chemiluminescence (ECL) system according to the manufacturer’s instructions. 

### Statistical analysis

Experiments were conducted independently at least twice in triplicate. Figures depict the results of an individual experiment (mean ± standard deviation, n = 3), which are representative for the repeat tests. A Student’s t-test was performed to determine differences between C3 toxin-treated and untreated cells. Statistical differences (SPSS, version 19; IBM, Ulm, Germany) of p < 0.05 were considered significant.

## References

[1] Aktories K, Frevert J (1987) ADP-ribosylation of a 21–24 kDa eukaryotic protein(s) by C3, a novel botulinum ADP-ribosyltransferase, is regulated by guanine nucleotide. Biochem J 247: 363–368. PubMed: 3122724. 3122724 10.1042/bj2470363 PMC1148417

[2] Just I, Mohr C, Schallehn G, Menard L, Didsbury JR et al. (1992) Purification and characterization of an ADP-ribosyltransferase produced by *Clostridium* *limosum* . J Biol Chem 267: 10274–10280. PubMed: 1587816. 1587816

[3] Vogelsgesang M, Pautsch A, Aktories K (2007) C3 exoenzymes, novel insights into structure and action of Rho-ADP-ribosylating toxins. Naunyn Schmiedeberg's Arch Pharmacol 374: 347-360. doi:10.1007/s00210-006-0113-y. 17146673

[4] Aktories K, Wilde C, Vogelsgesang M (2004) Rho-modifying C3-like ADP-ribosyltransferases. Rev Physiol Biochem Pharmacol 152: 1-22. PubMed: 15372308. 15372308 10.1007/s10254-004-0034-4

[5] Fahrer J, Kuban J, Heine K, Rupps G, Kaiser E et al. (2010) Selective and specific internalization of clostridial C3 ADP-ribosyltransferases into macrophages and monocytes. Cell Microbiol 12: 233-247. doi:10.1111/j.1462-5822.2009.01393.x. PubMed: 19840027. 19840027

[6] Aktories K, Jung M, Böhmer J, Fritz G, Vandekerckhove J et al. (1995) Studies on the active site structure of C3-like exoenzymes: involvement of glutamic acid in catalysis of ADP-ribosylation. Biochimie 77: 326–332. doi:10.1016/0300-9084(96)88142-9. PubMed: 8527485. 8527485

[7] Lillich M, Chen X, Weil T, Barth H, Fahrer J (2012) Streptavidin-conjugated C3 protein mediates the delivery of mono-biotinylated RNAse A into macrophages. Bioconjugate Chem 23: 1426-1436. doi:10.1021/bc300041z. 22681511

[8] Dmochewitz L, Förtsch C, Zwerger C, Väth M, Felder E et al. (2013) A recombinant fusion toxin based on enzymatic inactive C3bot1 selectively targets macrophages. PLOS ONE 8: e54517. doi:10.1371/journal.pone.0054517. PubMed: 23349915. 23349915 PMC3549961

[9] Barth H, Hofmann F, Olenik C, Just I, Aktories K (1998) The N-terminal part of the enzyme component (C2I) of the binary *Clostridium* *botulinum* C2 toxin interacts with the binding component C2II and functions as a carrier system for a Rho ADP-ribosylating C3-like fusion toxin. Infect Immun 66: 1364-1369. PubMed: 9529054. 9529054 10.1128/iai.66.4.1364-1369.1998 PMC108061

[10] Aktories K, Barth H (2011) New insights into the mode of action of the actin ADP-ribosylating virulence factors Salmonella enterica SpvB and Clostridium botulinum C2 toxin. Eur J Cell Biol 90: 944-950. doi:10.1016/j.ejcb.2010.11.007. PubMed: 21247657. 21247657

[11] Barth H, Stiles BG (2008) Binary actin-ADP-ribosylating toxins and their use as Molecular Trojan Horses for drug delivery into eukaryotic cells. Curr Med Chem 15: 459-469. doi:10.2174/092986708783503195. PubMed: 18289001. 18289001

[12] Yavropoulou MP, Yovos JG (2008) Osteoclastogenesis - current knowledge and future perspectives. J Musculoskelet Neuronal Interact 8: 204-216. PubMed: 18799853. 18799853

[13] Saltel F, Chabadel A, Bonnelye E, Jurdic P (2008) Actin cytoskeletal organisation in osteoclasts: a model to decipher transmigration and matrix degradation. Eur J Cell Biol 87: 459-468. doi:10.1016/j.ejcb.2008.01.001. PubMed: 18294724. 18294724

[14] Zhang D, Udagawa N, Nakamura I, Murakami H, Saito S et al. (1995) The small GTP-binding protein, rho p21, is involved in bone resorption by regulating cytoskeletal organization in osteoclasts. J Cell Sci 108: 2285-2292. PubMed: 7673348. 7673348 10.1242/jcs.108.6.2285

[15] Chellaiah MA (2005) Regulation of actin ring formation by Rho GTPases in osteoclasts. J Biol Chem 23: 32930-32943. PubMed: 16006560. 10.1074/jbc.M500154200 16006560

[16] Itzstein C, Coxon FP, Rogers MJ (2011) The regulation of osteoclast function and bone resorption by small GTPases. LANDES Bioscience 2: 117-130. 10.4161/sgtp.2.3.16453 PMC3136942 21776413

[17] Brazier H, Stephens S, Ory S, Fort P, Morrison N et al. (2006) Expression profile of RhoGTPases and RhoGEFs during RANKL-stimulated osteoclastogenesis: identification of essential genes in osteoclasts. J Bone Miner Res 21: 1387-1398. doi:10.1359/jbmr.060613. PubMed: 16939397. 16939397

[18] Hu Y, Nyman J, Muhonen P, Väänänen HK, Laitala-Leinonen T (2005) Inhibition of the osteoclast V-ATPase by small interfering RNAs. FEBS Lett 579: 4937-4942. doi:10.1016/j.febslet.2005.07.078. PubMed: 16115623. 16115623

[19] Wang Y, Tran KK, Shen H, Grainger DW (2012) Selective local delivery of RANK siRNA to bone phagocytes using bone augmentation biomaterials. Biomaterials 33: 8540-8547. doi:10.1016/j.biomaterials.2012.07.039. PubMed: 22951320. 22951320 PMC3444628

[20] Christow H, Lillich M, Sold A, Fahrer J, Barth H (2013) Recombinant streptavidin-C3bot for delivery of proteins into macrophages. Toxicon. doi: 10.1016/j.toxicon.2013.02.002. 23422352

[21] Agholme F, Andersson T, Tengvall P, Aspenberg P (2012) Local bisphosphonate release versus hydroxyapatite coating for stainless steel screw fixation in rat tibiae. J Mater Sci Mater Med 23: 743-752. doi:10.1007/s10856-011-4539-5. PubMed: 22203517. 22203517

[22] Hsu H, Lacey DL, Dunstan CR, Solovyev I, Colombero A et al. (1999) Tumor necrosis factor receptor family member RANK mediates osteoclast differentiation and activation induced by osteoprotegerin ligand. Proc Natl Acad Sci U S A 96: 3540-3545. doi:10.1073/pnas.96.7.3540. PubMed: 10097072. 10097072 PMC22329

[23] Alley MC, Scudiero DA, Monks A, Hursey ML, Czerwinski MJ et al. (1988) Feasibility of drug screening with panels of human tumor cell lines using a microculture tetrazolium assay. Cancer Res 48: 589-601. PubMed: 3335022. 3335022

[24] Laemmli UK (1970) Cleavage of structural proteins during the assembly of the head of bacteriophage T4. Nature 227: 680-685. doi:10.1038/227680a0. PubMed: 5432063. 5432063

